# Drug‐induced liver injury from Deferasirox in a pediatric patient with hereditary spherocytosis: A case report

**DOI:** 10.1002/jpr3.12155

**Published:** 2024-12-16

**Authors:** Siddhant Talwar, Blake Rosenthal, Madhav Vissa, Kayla Cort, Joshua Byers, Sabina Ali

**Affiliations:** ^1^ Department of Pediatrics UCSF Benioff Children's Hospital‐Oakland Oakland California USA; ^2^ Division of Pediatric Gastroenterology, Department of Pediatrics Children's Hospital Los Angeles Los Angeles California USA; ^3^ Division of Pediatric Hematology, Department of Pediatrics UCSF Benioff Children's Hospital‐Oakland Oakland California USA; ^4^ Division of Pediatric Radiology, Department of Pediatrics UCSF Benioff Children's Hospital‐Oakland Oakland California USA; ^5^ Departments of Pathology and Laboratory Medicine UCSF San Fransisco California USA; ^6^ Division of Pediatric Gastroenterology, Department of Pediatrics UCSF Benioff Children's Hospital‐Oakland Oakland California USA

**Keywords:** anemia, chelation, hepatocellular injury, hyperbilirubinemia

## Abstract

Patients with hereditary spherocytosis (HS) often require red blood cell transfusions for the treatment of hemolytic anemia. Iron overload is a known complication of frequent transfusions. Deferasirox, an oral iron chelator, can cause transient elevations in serum aminotransferase levels. There have been a few cases demonstrating Deferasirox‐associated liver injury in patients with sickle cell anemia and thalassemia. In this case report, we present a 13‐year‐old male with transfusion‐dependent HS treated with Deferasirox who presented with jaundice and was found to have evidence of acute hepatocellular injury.

## 1 INTRODUCTION

Hereditary spherocytosis (HS) is a congenital hemolytic anemia caused by abnormal red blood cell (RBC) membrane integrity.^1^ It is one of the most commonly inherited RBC membrane disorders. Splenomegaly, anemia, and unconjugated hyperbilirubinemia are common.

Treatment of HS involves symptomatic care, avoiding complications due to hemolysis and anemia, periodic RBC transfusions, and splenectomy.^2^ RBC transfusions can lead to iron overload (IO), for which the mainstay of treatment is chelation therapy. Deferasirox is an oral iron chelator that is primarily metabolized by hepatocytes and chelates tissue iron, eliminating it in bile.^3^ Deferasirox remains the first‐line agent for transfusional IO due to its ease of use and availability in oral tablet or sprinkle formulation. There is limited data indicating an association between Deferasirox and liver injury.

## 2 CASE REPORT

A 13‐year‐old African American male with a history of transfusion‐dependent HS and IO on chelation therapy with Deferasirox presented to the ED with jaundice and abdominal pain. He denied fever or new medications.

**Figure 1:**
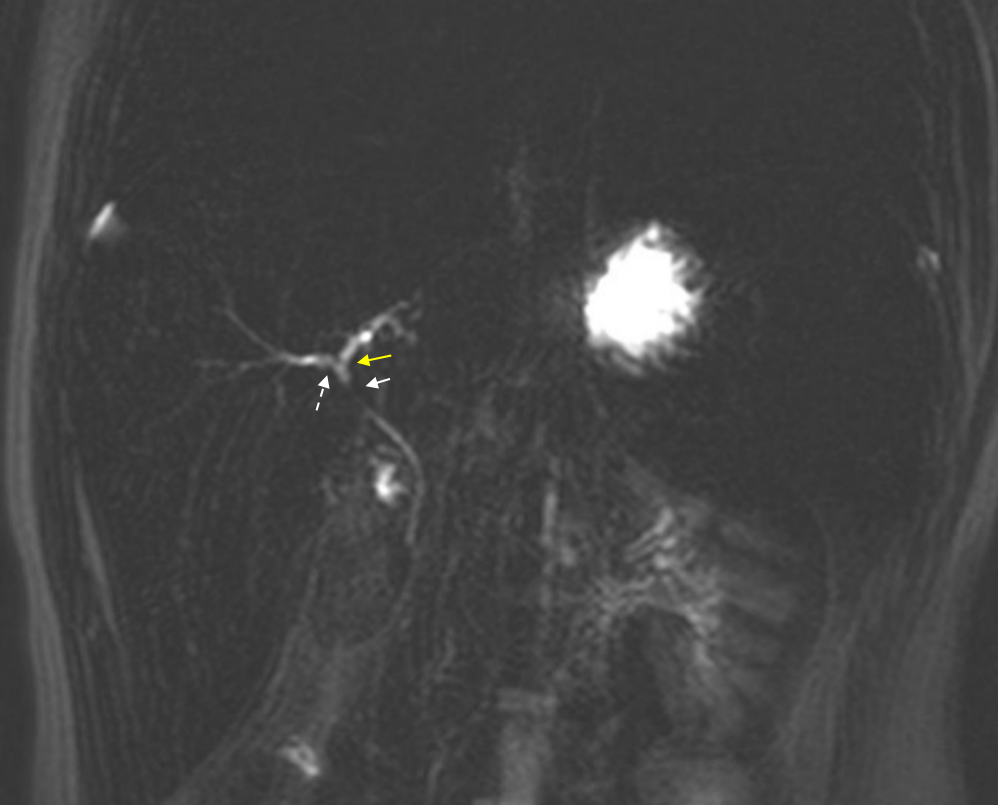
3D‐reconstructed MRCP demonstrates smooth narrowing of the common hepatic duct (solid white arrow), mild focal narrowing at the origins of the right hepatic duct (dashed arrow) and left hepatic duct (yellow arrow). 3D, three‐dimensional; MRCP, magnetic resonance cholangiopancreatography.

On presentation, he was mildly tachycardic with otherwise normal vitals. A physical exam showed jaundice, scleral icterus, and hepatomegaly. Splenomegaly was not appreciated on exam. Labs were notable for hemoglobin (Hb) 11.5 g/dL (12.5–16 g/dL), reticulocyte percentage 0.4% (0.5%–1.5%), aspartate transaminase (AST) 101 U/L (8–60 U/L), and alanine transaminase (ALT) 192 U/L (0–41 U/L). His baseline pre‐transfusion Hb was 7.5–8.5 g/dL. There was marked elevation in direct bilirubin (DB) >20 mg/dL (0–0.30 mg/dL) and total bilirubin (TB) 38.50 mg/dL (0–1.20 mg/dL). GGT 127 U/L (8–61 U/L). Alkaline phosphatase (AP) was 506 U/L (16–530 U/L), and international normalized ratio was 1.2 (0.8–1.2). Albumin was 4.8 g/dL (3.5–4.8 g/dL). Abdominal ultrasound (AUS) showed hepatosplenomegaly and cholelithiasis without cholecystitis. The common bile duct diameter was 1.7 mm. Magnetic resonance cholangiopancreatography (MRCP) showed cholelithiasis and nonspecific narrowing of the common hepatic duct thought to be related to crossing of the hepatic artery without dilatation of the biliary tree (Figure 1).

The *differential diagnosis* in this case is broad and includes anatomic, infectious, autoimmune, and drug‐induced liver injury (DILI). Although AUS showed cholelithiasis, there was no evidence of choledocholithiasis or biliary ductal dilation on MRCP. Testing for hepatitis A, B, and C was negative. Celiac and thyroid disease, screening was negative. Serum ceruloplasmin for Wilson's disease was normal, and alpha 1 antitrypsin phenotype was M1M1. In assessing for autoimmune hepatitis, the patient's anti‐smooth muscle antibody was weakly positive (1:80) with negative antinuclear antibody and nonreactive liver kidney microsome antibody. Hemolysis in the setting of known HS could cause unconjugated hyperbilirubinemia but would not explain CB. The patient's Hb also did not drop below expected for his degree of ongoing hemolysis, suggesting that accelerated hemolysis was not contributing substantially to his hyperbilirubinemia. Liver injury due to IO from chronic transfusions was also thought to be less likely as the patient's baseline liver enzyme testing was normal and liver iron content was relatively low at 2.1 mg/g dry weight liver. DILI was high up on the differential given the negative work‐up.

**Figure 2:**
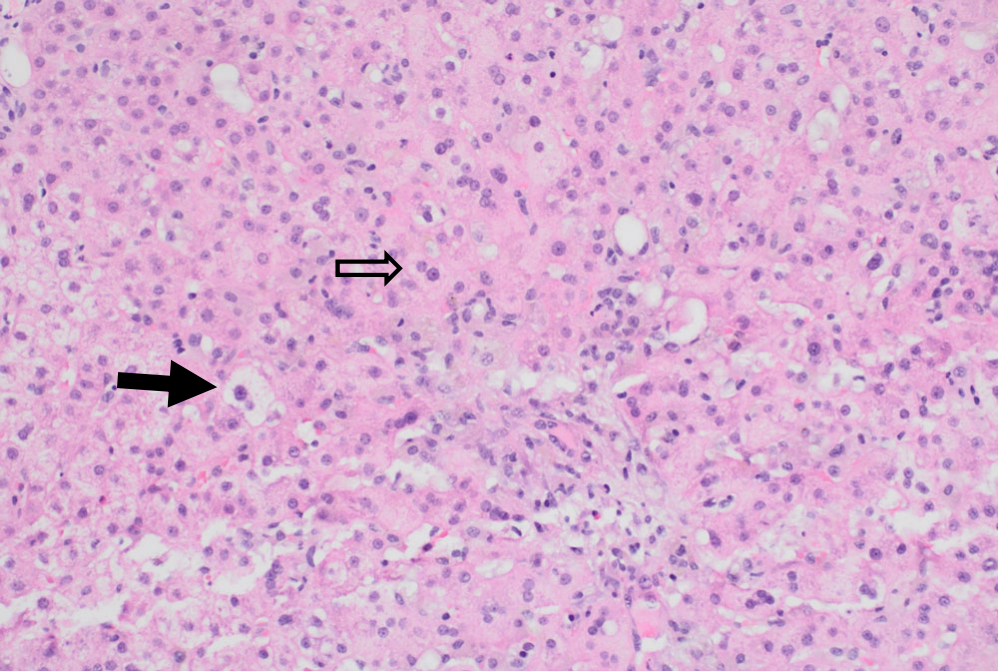
There is significant hepatocyte injury, including degenerating hepatocytes and ballooned hepatocytes (dark arrow). Regenerating hepatocytes with multiple nuclei are also noted (open arrow).

**Figure 3:**
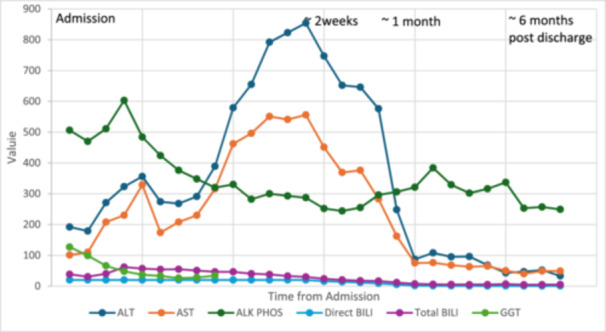
Graph of AST/ALT, direct bilirubin, total bilirubin, Alk Phos, and GGT from admission to 6 months post‐discharge. ALT, alanine transaminase; AST, aspartate transaminase; GGT, gamma‐glutamyltransferase.

AST 556 U/L (8–60 U/L) and ALT 854 U/L (0–41 U/L) peaked on Day 15 of admission. TB peaked at 61.87 mg/dL (0–1.20 mg/dL) on Day 3. AUS‐guided liver biopsy showed acute and subacute hepatopathy (Figure 2), which was thought to be secondary to Deferasirox. The medication was stopped on the day of admission and switched to intravenous Deferoxamine. DB and GGT slowly decreased after stopping Deferasirox. Two weeks after stopping Deferasirox, the patient's DB and TB levels were 13.98 mg/dL (0–0.3 mg/dL) and 20.17 mg/dL (0–1.20 mg/dL), respectively. GGT at that time was normal 34 U/L (8–61 U/L). Six months after discontinuation, his liver labs were close to normal with DB 0.62 mg/dL (0–0.3 mg/dL) and TB 5.17 mg/dL (0–1.20 mg/dL) with his AST/ALT 49 U/L (8–60 U/L)/33 U/L (0–41 U/L) (see Figure 3).

## 3 DISCUSSION

In this case report, we present a patient with HS who was found to have DILI without progression to fulminant liver failure in the setting of Deferasirox chelation therapy. His cholestasis and hepatitis improved after discontinuation of Deferasirox and remained controlled after initiation of other chelators.

The most commonly reported adverse reactions associated with Deferasirox include diarrhea, vomiting, abdominal pain, skin rashes, and increases in serum creatinine.^4^ Renal impairment from Deferasirox has been extensively described; however, there is relatively less data surrounding liver injury.^5^ Large pediatric studies have demonstrated that ~5% of patients on Deferasirox have transient elevation in serum aminotransferase levels above five times the upper limit of normal (ULN).^6^ There are reports of Deferasirox‐associated acute liver failure in pediatric and adult patients with other types of hemolytic anemia, such as sickle cell disease and thalassemia. The patients who did not recover had an additional risk for hepatotoxicity, either through genetic mutation, mitochondrial dysfunction, or active viral infection.^7^, ^8^, ^9^, ^10^, ^11^ No patients required liver transplantation. Although there are multiple etiologies of liver dysfunction in thalassemia major, our patient has nonthalassemia hemolytic anemia and may not have the same mechanism of liver injury.

DILI occurs when there is microscopic evidence of liver damage due to impaired clearance of metabolites in the setting of medications or herbal substances with an elevation in liver enzymes, including ALT, CB, or AP exceeding two times the ULN.^12^ The Roussel Uclaf Causality Assessment Method (RUCAM) scoring system can help evaluate the likelihood that a medication has caused DILI. The scoring criteria are represented as the following: <0, excluded; 1–2, unlikely; 3–5, possible; 6–8, probable; and >9, highly probable. The *R* ratio determines whether the injury is hepatocellular (*R* > 5.0), cholestatic (*R* < 2.0), or mixed (*R* = 2.0–5.0). For our patient, the *R* ratio was calculated to be 4.9, indicating a mixed hepatocellular/cholestatic picture. The RUCAM score for our patient was calculated to be 6 (1+ for “Time to onset,” 2+ for “Course,” 1+ for “Exclusion of other causes of liver injury,” and 2+ for “Previous information on hepatoxicity of the drug,” indicating probable DILI). DILI was later confirmed with biopsy, which showed a microscopic pattern of hepatocellular injury.

This case adds to existing literature describing DILI from an iron chelation therapy. Additional research is needed to better describe the exact mechanism of injury and for the development of preventative medications. Presently, routine monitoring of aminotransferase and fractionated bilirubin levels is recommended to prevent progression to liver injury.^13^

Following are the teaching points we would like to share through this case report:

1. The differential for elevated transaminases and cholestasis in a patient with HS requiring chronic transfusions is broad and includes hemolytic, anatomic, infectious, autoimmune, metabolic, and DILI.
2. Chronic transfusion‐related IO, as well as iron chelation therapy, can cause liver injury.
3. The RUCAM scoring system can be useful in determining the likelihood that a medication has caused DILI. However, biopsy remains the gold standard for diagnosis.

## ACKNOWLEDGMENTS

The authors have nothing to report.

## CONFLICT OF INTEREST STATEMENT

The authors declare no conflicts of interest.

## ETHICS STATEMENT

Written informed consent was obtained by the patient and family for this case report.
